# The Toxins of Pneumonia

**Published:** 1897-02-13

**Authors:** 


### The Toxins of Pneumonia.

The steadily increasing evidence which is accumulating in favour of the view that pneumonia is an infective disease much more allied to the acute fevers than to any mere local "inflammation," should make us reconsider many of the ideas which have held their ground for many years in regard to the meaning of its various symptoms and the treatment which they may require.

The morbid anatomy of pneumonia is strikingly suggestive of the disease being an exanthem affecting the enormous convoluted surface which lines the air cells. However extensive it may be, and however large a portion of the lung may be affected, we rarely find that the lung substance is permanently damaged, and, so soon as it is set free from the effusion in which it has been embedded, it returns to its old condition and takes on again its old activity.

Although pneumonia still retains its place in the text books next door to bronchitis and such like local ailments, it seems pretty certain that before long it will claim its proper place among the infections. The chief objection of the older school to giving to pneumonia such a place arises from the fact that, as they say, it is not generally an infectious disease. But then if one looks at infection in a limited sense, no more are cholera or typhoid fever, yet no one would deny to either of them the right to be looked on as infectious diseases; and when we once know the route by which the infection of pneumonia travels, we shall all doubtless admit its right to be looked on as a specific fever, and may perhaps be able to lessen the frequency of its occurrence.

Looked at in the light of modern knowledge, the various symptoms of pneumonia indicate the several steps of an infective process, one involving, as other infective processes do, the production of toxins, the poisoning of the tissues of the body far away from the apparent seat of the disorder, and probably the ultimate development of anti-toxins, by whose aid the malady is brought to an end as definite, and often as sudden as the defervescence in a case of typhus or of measles.

Dr. William Osler, writing in the *American Journal of the Medical Sciences*, has done well to draw attention to certain points in the prognosis of pneumonia, and especially to the fact that outside and beyond the effusion in the pulmonary tissues the symptoms are the result of a toxæmia, and that it is on the violence of this toxæmia, and the power of the patient to resist it, that the issue depends. It is the toxæmia, he insists, which is the important element in the disorder, and to this in the majority of cases the degree of pyrexia and the consolidation are entirely subsidiary. The signs of poisoning may develop early, and may from the outset cause severe cerebral complications. A patient with complete consolidation of one lobe, or of a whole lung, may have no delirium from the beginning to the end of his attack, while there may be fatal toxæmia with consolidation of only half a lobe, or in cases in which there may be no cough, no expectoration, very slight fever, and no leucocytosis. Most important of all, however, is it to bear in mind that the sudden and unexpected death which too often occurs in pneumonia is to be attributed to the action of specific toxins on the heart centres rather than to strain on the muscular substance of the heart itself.

Mechanical interference with the circulation is a much less frequent cause of death, nor must the dilatation of the cavities of the right side of the heart, which occurs in such cases, be looked on as produced entirely as the result of physical obstruction in the lung. Very large areas of the breathing surface may be cut off without seriously disturbing the cardio-respiratory mechanism, while, on the other hand, cases in which the interference with the circulation through the lung had seemed profound may, immediately after the crisis, become free from dyspnœa and cyanosis, although the physical condition of the lung may remain the same. The toxæmia, says Dr. Osler, outweighs all other elements in the prognosis of pneumonia; to it is due in great part the terrible mortality from this common disease, and, unhappily, against it we have as yet no reliable measures at our disposal. Earnest men, however, are working at anti-toxins, and we must not despair. How many cases of pneumonia die apparently almost within touch of the crisis! An anti-toxin which could hasten matters and make the tide turn sooner, if even by but a few hours, might save an enormous number of lives.

